# A rare cardiac inflammatory myofibroblastic tumor involving aortic valve

**DOI:** 10.1186/s13019-017-0577-5

**Published:** 2017-03-02

**Authors:** Wenzong Luo, Peng Teng, Yiming Ni

**Affiliations:** 1 Department of Cardiothoracic Surgery, The First Affiliated Hospital of Zhejiang University, Hangzhou, China; 2 79#, Qingchun Road, Hangzhou, Zhejiang 310000 China

**Keywords:** Inflammatory myofibroblastic tumor, Aortic valve, Middle age

## Abstract

**Background:**

Cardiac inflammatory myofibroblastic tumor (IMT) is an extremely rare benign entity which constitutes a small proportion of primary cardiac tumor.

**Case presentation:**

We present a rare case of a 55-year-old symptomatic male patient with a rare cardiac IMT causing left ventricular outflow tract (LVOT) obstruction. The patient received complete tumor resection immediately with uneventful postoperative hospital course.

**Conclusion:**

To our best knowledge, cardiac IMT involving aortic valve in older adult causing LVOT obstruction has never been reported before. Additionally, we make a literature review on IMT, focusing on the various clinical presentation of this unpredictable tumor.

## Background

Primary cardiac tumors are rare diseases with an autopsy incidence ranging from 0.0017 to 0.27%. Myxomas and sarcomas are the most common benign and malignant cardiac neoplasms, respectively. IMT, previously termed as plasma cell granuloma or inflammatory pseudotumor, was first described in lung in 1939 [1]. IMTs most frequently occur in soft tissues and are composed predominantly of differentiated myofibroblastic cells accompanied by inflammatory mononuclear cells. Cardiac IMT is extremely rare with unknown etiology, mainly seen in children and young adults. To our best knowledge, less than 50 cases about cardiac IMTs have been published in English over recent decades. Herein, we present a middle aged male patient with cardiac IMT involving aortic valve, which caused LVOT obstruction.

## Case Presentation

**Fig. 1:**
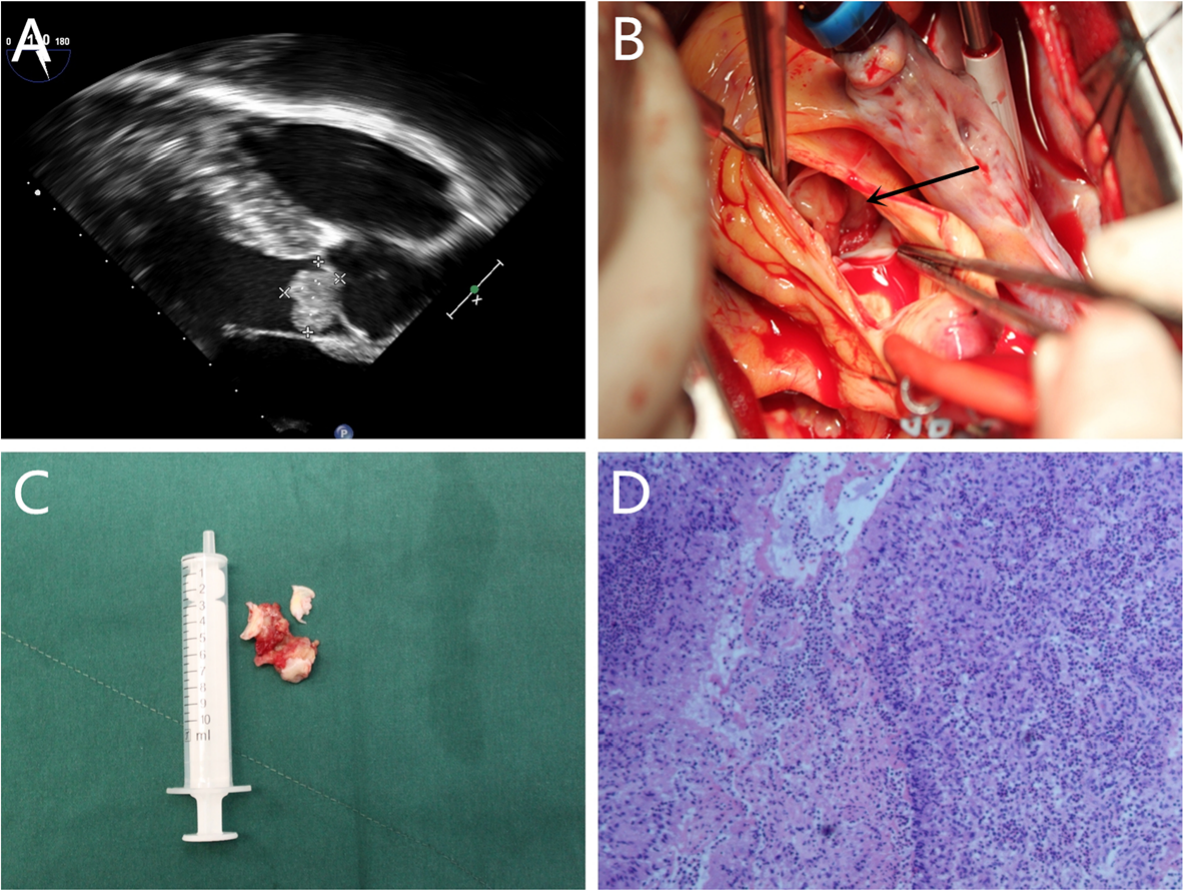
**a** Transthoracic echocardiography showed a well-defined homogenous hyperechoic mass (2.1 × 1.9 × 1.6 cm) at the LVOT. **b** An aortotomy revealed a mass (in *black arrow*) locating at the LVOT, inferior to the aortic valve. **c** The non-coronary cusp and right coronary cusp of the aortic valve was involved by the tumor. The tumor was totally resected and the aortic valve was removed. **d** Pathological study of the tumor showed severe proliferation of the myofibroblastic cells, surrounded by suppurative inflammatory tissues and extensive formation of granulomas which confirmed the diagnosis of inflammatory myofibroblastic tumor (Hematoxylin & eosin, magnification, ×40). (LVOT = Left ventricular outflow tract)

A 55-year-old Chinese male, with no contributory medical and family history, was admitted to our hospital because of exertional chest pain for 10 days. Physical examination revealed a systolic murmur (Grade II) in the left third intercostal space. Laboratory tests were unremarkable. The transthoracic echocardiography showed a pedunculated hyperechoic mass (2.1 × 1.9 × 1.6 cm) at LVOT, with a 0.5 cm pedicle adhere to the interventricular septum (Fig. 1a). The transvalvular blood flow velocity through aortic valve on Doppler mode was 4 m/s, indicating severe LVOT obstruction caused by the mass. Further assessment by computed tomographic angiography of coronary artery showed a spherical high density mass, which located at the LOVT, without coronary artery disease.

Tumor resection was performed via median sternotomy under general anesthesia and the cardiopulmonary bypass was established with aortic and right atrial cannulation. An aortotomy revealed a mass, about 3 × 2.5 cm in size, locating at the LVOT inferior to the aortic valve with a pedicle, about 0.5 cm in diameter, rising from the interventricular septum (Fig. 1b). In addition, the aortic valve was found extensively invaded by the tumor, especially the right coronary cusp and the non-coronary cusp. Simply tumor resection seemed impossible owing to the tumor location and widely involvement. Finally, the patient received complete tumor resection as well as aortic valve replacement with a 23 cm mechanical valve (Carbomedics, Sorin Group, Italy) (Fig. 1c). The pathological examination confirmed the diagnosis of IMT (Fig. 1d). The patient had an uneventful recovery without any complications and he was discharged home on the 7^th^ postoperative day. The patient has been followed up for 2 years without any sign of recurrence.

## Discussion

IMTs occur mainly in the soft tissues and are composed with predominantly of inflammatory and myofibroblastic cells. It is Brunn who described the first case of IMT in the lungs in 1939 [1]. Although it has been described in various lesions, cardiac lesions are much more uncommon. With our best knowledge, less than 50 cases of cardiac IMTs have been published in English since the initial description made by Gonzalez-Crussi in 1975 [2]. Owing to its extremely rarity, the etiology of IMT still remains unresolved and the immunologic and infectious postulates are still to be validated.

According to our literature review, it may seem premature to establish an epidemiologic profile of cardiac IMT owing to its rarity, but several features are concluded: no gender predominance, predilection for children and young adult. More than two thirds of the cardiac IMT cases have been reported in children and adolescents. To our best knowledge, less than 10 cases of cardiac IMT involving older adult patients have been published before, which makes our case much rarer.

Histologically, IMT consists of a proliferation of spindle-shaped cells associated with no remarkable atypia or mitotic activity, corresponding to fibroblasts and myofibroblasts arranged in a myxoid stroma made of a diffuse inflammatory infiltration with a heterogeneous cell admixture, dominated by histiocytes and plasmocytes. Immunohistochemical analysis is highly contributive to the differential diagnosis, objectifying universally positive for vimentin, universally negative for CD34 and generally positive for CD68 and smooth muscle actin antibodies [3].

The clinical presentation of IMT depends on multiple factors including patient age, tumor size, location, rate of growth and individual tolerance. Patients with cardiac IMT are usually asymptomatic until hemodynamic changes and local invasion leading to cardiac insufficiency. Decreased exercise tolerance may be the most common symptom in the patients with cardiac IMT. Arrhythmia may emerge when the conduction system is involved or ectopic excitation foci occurs. Once the valves are involved, symptoms related to valvular stenosis or regurgitation like lower extremity edema, dyspnea, syncope, chest pain may manifest. Moreover, fever of unknown origin is another possible symptom of IMT which suggested the nature of immunological-related.

The natural history of IMT is unpredictable. Patients undergoing surgical resection of the tumor usually have a favorable prognosis, while patients with an unresectable tumor may have a poor one because of the unpredictable progression of the tumor, like sudden death or embolism. Spontaneous or steroid –induced regression have also been reported in cardiac IMT [4, 5].

There is consensus about surgical treatment in symptomatic patients with cardiac IMT [6], but it is still controversial whether performing surgery in asymptomatic patients. Recurrence of IMT is more likely to occur in patients with incomplete tumor resection. No distant metastasis has been documented.

## Conclusion

Cardiac IMT is an extremely rare entity which constitutes only a few percent of primary cardiac tumors. This case report describes a rare cardiac IMT involving aortic valve in an older adult patient which has seldomly been reported before. Its etiology still remains controversy. Current consensus regarding optimal treatment for IMT is complete tumor resection which is associated with favorable long-term outcomes.

## Abbreviations

IMT: Inflammatory myofibroblastic tumor
LVOT: Left ventricular outflow tract
